# Unconventional Secretion of Nigerolysins A from *Aspergillus* Species

**DOI:** 10.3390/microorganisms8121973

**Published:** 2020-12-11

**Authors:** Nada Kraševec, Maruša Novak, Simona Barat, Matej Skočaj, Kristina Sepčić, Gregor Anderluh

**Affiliations:** 1 National Institute of Chemistry, SI-1000 Ljubljana, Slovenia; simona.barat@gmail.com (S.B.); gregor.anderluh@ki.si (G.A.); 2 Department of Biology, Biotechnical Faculty, University of Ljubljana, SI-1000 Ljubljana, Slovenia; marusa.novak68@gmail.com (M.N.); skocaj.matej@gmail.com (M.S.); Kristina.Sepcic@bf.uni-lj.si (K.S.)

**Keywords:** aegerolysin, nigerolysin, membrane-attack-complex/perforin (MACPF) protein, *Aspergillus*, Fungi, bioinformatics, localization, unconventional secretion, ceramide phosphoethanolamine (CPE), invertebrate lipid binding

## Abstract

Aegerolysins are small lipid-binding proteins particularly abundant in fungi. Aegerolysins from oyster mushrooms interact with an insect-specific membrane lipid and, together with MACPF proteins produced by the same organism, form pesticidal pore-forming complexes. The specific interaction with the same membrane lipid was recently demonstrated for nigerolysin A2 (NigA2), an aegerolysin from *Aspergillus niger*. In *Aspergillus* species, the aegerolysins were frequently found as secreted proteins, indicating their function in fungal defense. Using immunocytochemistry and live-cell imaging we investigated the subcellular localization of the nigerolysins A in *A. niger*, while their secretion was addressed by secretion prediction and Western blotting. We show that both nigerolysins A are leaderless proteins that reach the cell exterior by an unconventional protein secretion. NigA proteins are evenly distributed in the cytoplasm of fungal hyphae. A detailed bioinformatics analysis of *Aspergillus* aegerolysins suggests that the same function occurs only in a limited number of aegerolysins. From alignment, analysis of chromosomal loci, orthology, synteny, and phylogeny it follows that the same or a similar function described for pairs of pesticidal proteins of *Pleurotus* sp. can be expected in species of the subgenus *Circumdati*, section *Nigri*, series *Nigri*, and some other species with adjacent pairs of putative pesticidal proteins.

## 1. Introduction

Members of the aegerolysin protein family (PF06355; InterPro IPR009413) are acidic, β-structured, and low molecular weight (13–20 kDa) and have been found in various eukaryotic and bacterial taxa and are particularly abundant in Fungi [1,2,3]. The common feature of aegerolysins is their interaction with specific lipids in biological and artificial membranes [4,5,6,7]. While the exact biological role of these proteins in fungi is slowly becoming clear, some potential biotechnological applications have already become apparent [2,3]. For example, it has been reported that a promoter from a hemolysin-like gene (*hlyA*) encoding for an aegerolysin-like protein in *Aspergillus oryzae* [8] efficiently overexpressed several homologous and heterologous genes; expression levels were higher than when under the control of the commonly used *amyA* promoter [9]. Furthermore, an additional strong, small, constitutive promoter from the *A. niger* hemolysin gene (or a truncated variant thereof) was reported to have promoter activity in several fungal host cells [10]. Due to their ability to target sphingomyelin/cholesterol membrane domains [4,5,6,11,12], aegerolysins from the Basidiomycetes genus *Pleurotus* have been proposed as non-toxic biomarkers for functional and structural studies of membrane rafts in living cells [6,12]. These aegerolysins, especially ostreolysin A6 (OlyA6) and pleurotolysin A (PlyA) from *Pleurotus ostreatus*, can form bi-component pore-forming complexes in combination with pleurotolysin B (PlyB), a 59-kDa, membrane-attack-complex/perforin (MACPF)-like protein (PF01823) produced by the same mushroom [5,11,13,14]. OlyA6/PlyB protein complexes can be used to selectively kill cancer cells with elevated levels of membrane sphingomyelin/cholesterol [15]. Recently, *Pleurotus* aegerolysins have also been shown to interact with the ceramide phosphoethanolamine (CPE) [7,16,17], the major membrane sphingolipid of invertebrates, especially insects and mollusks [18]. This interaction, which is three orders of magnitude stronger than the binding to sphingomyelin/cholesterol membrane complexes [7], is responsible for the selective toxicity of aegerolysin-based protein complexes from *Pleurotus* mushrooms to two major insect pests: the larvae or adult Western corn rootworm and the larvae of the Colorado potato beetle [17], suggesting their use as environmentally friendly insecticides.

Fungi and fungal biotechnology have a great influence on our daily life [19,20]. Especially the fungal genus *Aspergillus* is important for mankind as a unique cell factory with an exceptionally high secretion capacity for enzymes of many industrial applications, but at the same time, it is also a source of important pathogens of humans, animals and crops, potent carcinogenic food contaminants, and an important genetic model [21]. Over 446 species of *Aspergillus* are already known [22]. The genome sequences of aspergilli have been explored for many years and many new ones will soon be revealed, mainly thanks to major sequencing efforts such as the 1000 Fungal Genomes Project or 300 *Aspergillus* genome project [21,22,23,24,25,26]. Well-developed genomics allows comparisons in terms of biology, evolution and specialization of fungi and shows high biodiversity and specific adaptations within the genus [21]. *Aspergillus* fungi have a high occurrence of aegerolysin proteins, and indeed these appear to be among the major proteins secreted by these fungi [16]. To date, over 328 putative aegerolysin sequences in over 226 genomes of sequenced *Aspergillus* species can be expected [23].

The first reported aegerolysin protein, Asp-hemolysin (AspHS), was isolated from the filamentous fungus *A. fumigatus*, the main cause of invasive aspergillosis in immunocompromised patients [27]. AspHS was initially reported to be hemolytic and involved in the pathogenesis of *A. fumigatus* [28]. However, wild-type and knock-out *aspHS* and *aspHS*-like mutant strains of *A. fumigatus* have shown that production of AspHS is not important for the progress of invasive alveolar aspergillosis in mice [29]. Instead, AspHS has been attributed to a role in potentiating other virulence mechanisms of the fungus [30]. Other published examples of aegerolysins in aspergilli are terrelysin from *A. terreus* [31] and AspHS homologs from *A. niger* [32,33].

The genome of *A. niger* contains two aegerolysin genes, *nigA1* and *nigA2*, and two MACPF-like genes, *nigB1* and *nigB2*. Following the nomenclature of aegerolysins in *Pleurotus* sp. we have named these proteins nigerolysins, NigA1 or NigA2 for aegerolysins and NigB1 or NigB2 for MACPF-like proteins [34]. In a previous study, we performed functional studies on the expression of the nigerolysin genes in *A. niger* [34]. A systematic study of the expression of genes encoding these proteins suggested that the sporulation process is crucial for a strong induction of the expression of all these genes. On the other hand, in the construction of *nigA* gene deletion mutants, the deletion of either of the *nigA* genes had no effect on the growth, development, sporulation efficiency, and phenotype of the mutants, suggesting that the nigerolysins are not key factors in the sporulation process. In all our expression studies a strong correlation was found in the expression of a one *nigA* and MACPF-like gene. Furthermore, the secretion of nigerolysins A from colonies was confirmed, which was consistent with previous transcriptomics or proteomics reports on protein secretion in *A. niger* under different growth conditions [35,36]. Our results thus confirmed previous findings that aegerolysin NigA2 is secreted from the fungus *A. niger*, although no signal peptide predicted in silico was proposed [32,33,37]. Other previously reported aegerolysins, AspHS from the fungus *A. fumigatus* and terrelysin from the fungus *A. terreus* have also been shown to be secreted [29,38]. Furthermore, the lipid-binding specificity of a recombinant *A. niger* NigA2 was analyzed. A specific interaction with an invertebrate-specific membrane sphingolipid CPE was detected, but in contrast to OlyA6, no interactions with sphingomyelin/ cholesterol were observed [34]. In addition, recombinant mCherry-labeled nigerolysin A2 stained insect cell membranes containing CPE. These data suggest that the physiological role of aegerolysins in this species and probably in other aspergilli in the extracellular environment may involve interactions with other (micro)organisms, especially predatory insects, and mollusks [34].

In this work, we performed an in-depth bioinformatics analysis of the aegerolysins in Fungi, with special emphasis on the genus *Aspergillus*. The first aim was to uncover the ability of fungi to successfully secrete aegerolysins, while the second aim was to broaden the genus-wide view of the biological diversity of these proteins in aspergilli. We approached the topics by theoretically predicting the secretion process together with live-cell imaging to reveal the localization of nigerolysins in the fungus *A. niger*. The understanding of the secretion ability of fungi is particularly important with regard to the interaction by binding to major sphingolipid CPE present in the membranes of invertebrate predators. 

## 2. Materials and Methods

### 2.1. Strains, Growth Conditions, and Chemicals

Fungal spores or mycelia were stored over the long term at −80 °C in 20% or 25% (*v*/*v*) glycerol, respectively. The plasmids were multiplied in *Escherichia coli* DH5α cells and isolated from them. The chemicals used were from Merck KGaA (Darmstadt, Germany) or Sigma-Aldrich (St. Louis, MO, USA) unless otherwise indicated. For spore preparation, the *Aspergillus* strains were grown in the dark on potato dextrose agar plates (Difco, Detroit, MI, USA) or on solid minimal medium (MM) with 1% (*w*/*v*) glucose, at 30 °C for four days [39]. The spores were collected in sterile 0.9% (*w*/*v*) NaCl and then stored at 4 °C for not more than three months. 

### 2.2. Preparation of Fluorescent Protein Cassettes for Fungal Transformation

**Figure 1:**
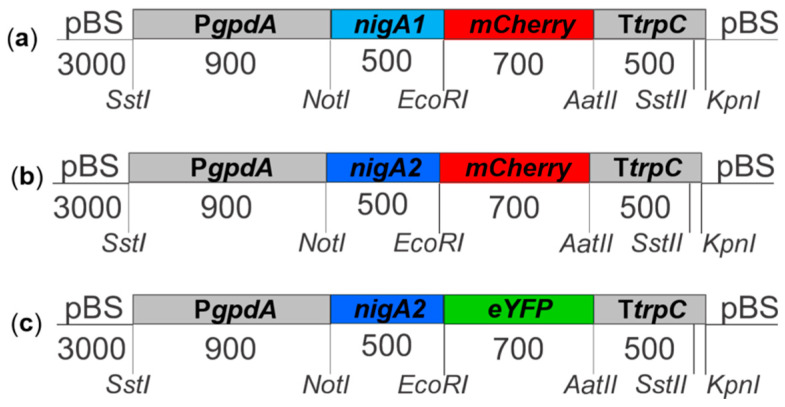
Schematic representation of expression cassettes for the transformation of the fungus *A. niger* strain N402. (**a**) *nigA1*-*mCherry*; (**b**) *nigA2-mCherry*; and (**c**) *nigA2-eYFP*. Restriction enzyme cleavage sites are indicated. P*gpdA*, glycerol phosphate dehydrogenase A gene promoter from *A. nidulans*; T*trpC*, tryptophan synthase C gene terminator from *A. nidulans*; *nigA1*, nigerolysin gene A1; *nigA2*, nigerolysin gene A2; *mCherry*, gene for red fluorescent protein; *eYFP*, gene for enhanced yellow fluorescent protein; pBS, plasmid pBlueScript (Stratagen, USA). The numbers of base pairs indicate the size of the individual parts.

Two variants of genes for fluorescent proteins (FP), the gene for the enhanced yellow fluorescent protein (eYFP) or the mCherry gene, were subcloned into the pMOJ009 vector [40] using the restriction sites *Nde*I and *Eco*RI, where, under the control of the strong constitutive glyceraldehyde phosphate dehydrogenase A promoter (P*gpdA*) and the tryptophan synthase C transcription terminator (T*trpC*) from *A. nidulans*, was expressed. The *nigA* genes without a translational stop codon were amplified from the genomic DNA of *A. niger* N402 [41] by pairs of the oligonucleotides *nigANot*IF and *nigAEco*RIR (Eurofins MWG Operon, Ebersberg, Germany) (Table 1) using the polymerase chain reaction (PCR) (KOD Hot Start DNA polymerase). The resulting PCR fragment and plasmid were cut with the restriction endonucleases *Not*I and *Eco*RI (New England Biolabs Inc., Ipswich, MA, USA) and ligated by ligase T4 (Biolabs) to obtain the plasmid pMOJ/ nigA-FP. The schematic representation of the expression cassettes for the transformation is shown in Figure 1.

### 2.3. Fungal Transformation, Selection, and Confirmation of Transformants

The *A. niger* strain N402 [41] was co-transformed with the protoplast method [39], with the *Kpn*I linearized plasmid pMOJ/nigA-FP and in parallel with the plasmid pAN7-1, which contains a gene with resistance to hygromycin B. The transformants were selected using selective MM with glucose containing 1.2 M sorbitol and hygromycin B (Sigma-Aldrich) (100 μg/mL) and UV light [42]. After an initial selection by UV light, the individual colonies were first inoculated twice on selective MM agar plates with glucose and hygromycin B and then on solid MM with glucose, from where the spores were collected and stored. Permanent cultures of the transformants NigA1-mCherry, NigA2-eYFP, and NigA2-mCherry were prepared from the spores. The success of the co-transformation was confirmed by observing the preparation of live mycelium with an inverted confocal microscope.

### 2.4. Construction of Nigerolysin Gene Deletion Mutants

The nigerolysin gene-deletion mutants of *A. niger* N402, named ∆*nigA1* and ∆*nigA2*, were constructed as described by Novak et al., 2019 [34]. Essentially, 5′ and 3′ sequences of the *nigA* genes from the genomic DNA of the wild-type strain *A. niger* N402 [41] were amplified by PCR, cut with the restriction enzymes *Not*I and *Xba*I or with *Hind*III and *Kpn*I, respectively, and inserted between *Xba*I and *Bam*HI sites of the plasmid pBlueScript on both sides of the hygromycin B resistance cassette (*hph*) from the plasmid pAN7-1 [39], which was previously inserted into the polylinker site [43]. The transformants were selected on the solid MM with 1% (*w*/*v*) glucose, 1.2 M sorbitol, and 100 µg/mL hygromycin B (Invivogen Corp., San Diego, CA, USA). The deletions of the two *nigA* genes were confirmed by two-step PCR and quantitative reverse transcription polymerase chain reaction (RT-qPCR).

### 2.5. Secretion of Nigerolysins A

To monitor the secretion of nigerolysins during fungal growth and development in liquid medium, strains *A. niger* N402, ∆*nigA1*, and ∆*nigA2* were cultivated as described previously [44]. In short, 10^8^ spores were inoculated into the complete medium and cultivated overnight at 30 °C with stirring at 180 rpm, then the biomass was transferred to MM with 1% (*w*/*v*) glucose where the fungus was cultivated for 24, 48, or 72 h. Media (<100 mL) was removed from the mycelia by filtration and concentrated to about 500 µL by centrifugal filtration (NMWL10; Amicon Ultra 15, Merck, DE). The media was separated on NuPAGE 4–12% Bis-Tris protein gel (Thermo Fisher Scientific Inc., Waltham, MA, USA) and transferred to PVDF membrane by iBlot Gel Transfer Device (Thermo Fisher Scientific Inc., Waltham, MA, USA), all according to the manufacturer instructions. Nigerolysins A on the PVDF membranes were detected by Western blotting. The PVDF membranes were first blocked for one hour with 5% skimmed milk in Tris-buffered saline solution, pH 7.4, and 0.1% Tween (TBS-T). The PVDF membranes were then incubated overnight at 4 °C with primary polyclonal mouse antibodies against recombinant NigA2 (1:1000; Department of Animal Science, Biotechnical Faculty, University of Ljubljana, Slovenia) [34], prepared in 5% skimmed milk in TBS-T. This was followed by a 2-h incubation at room temperature with the secondary monoclonal goat anti-mouse antibodies (1:2000; Sigma-Aldrich, USA), also prepared in 5% skimmed milk in TBS-T. Washing of the membranes between incubation times was performed with TBS-T. The proteins were detected with the enhanced chemiluminescence method (Pierce ECL Western Blotting substrate; Thermo Fisher Scientific Inc., Waltham, MA, USA), according to the manufacturer’s protocol. 

### 2.6. Live-Cell Imaging

Live-cell imaging techniques are now routinely used in studies of filamentous fungi, which has been facilitated by the development of a wide range of microscope technologies and fluorescence probes (vital dyes and FP) [45]. Images of the live fungal mycelium were obtained with the Leica TCS SP5 laser-scanning microscope on a Leica DMI 6000 CS inverted microscope (Leica Microsystems GmbH, Wetzlar, Germany) with an HCX plan apo x63 (numerical aperture, 1.4) oil immersion objective. To record fluorescence within the hyphae, 100 µL drops MM were incubated with *A. niger* NigA-FP conidia (10^5^ spores/mL) on a cover glass in a humid chamber for 18–24 h. During this time the conidia germinated and mycelia formed [40]. The mycelia were stained with different dyes/FP. Their selectivity in the cell compartment, working concentration, incubation time, absorption/emission (nm) is described in Table 2.

### 2.7. Immunolocalization and Cell Imaging

The *A. niger* N402 wild type cells were harvested from solid MM, covered with a porous cellophane membrane, embedded in optimal cutting temperature compound (OCT; Tissue Tek, Sakura Finetek Europe B.V., Alphen aan den Rijn, The Netherlands) and frozen in a cryostat Leica CM3000, (Leica Microsystems GmbH, Wetzlar, Germany) at −30 °C and cut into 10 µm-thick cryosections. The sections were fixed with 2% paraformaldehyde for 20 min at 22 °C. After washing with PBS (pH 7.4) at 22 °C the cells were blocked with 1 % BSA in PBS. The sections were incubated with anti-mouse NigA2 antibodies (1:1000 in 1% BSA in PBS) [34] for 2 h at 37 °C and then washed with PBS. After washing, the sections were incubated overnight with secondary goat anti-mouse-Alexa-488 antibodies (1:400) (Molecular Probes, Thermo Fisher Scientific Inc., Waltham, MA, USA) in 1% BSA in PBS, at 4 °C. The next day, the sections were washed with PBS and mounted in Vectashield for nuclear staining with 4′,6-diamidino-2-phenylindole (DAPI). Immunostained embedded cryoresin slides were observed with the inverted confocal microscope as described above. 

### 2.8. Comparative Genomics, Prediction, and Phylogenetic Methods

Mining for fungal aegerolysins was performed in several fungal databases, including: MycoCosm at Joint Genome Institute (JGI) [23]; The *Aspergillus* Genome Database (AspGD) [46]; Ensembl Fungi (EMBL-EBI) [47]; The Central *Aspergillus* Data Repository (CADRE) [48]; and FungiDB release 48 beta [49,50]. The databases were searched for the PF06355 domain named aegerolysin and protein sequences of pleurotolysin A from *P. ostreatus* and two sequences from *A. niger*, which code for nigerolysins, *nigA1* (An19g00210/ Aspni7|1145225) and *nigA2* (An01g09980/ Aspni7|1162650) and hits were combined. Hits from only one strain per species were considered for inclusion in the study data set, as proteins from different strains may have some point mutations (e.g., as in different strains of *P. ostreatus*), with the exception of the former species of *A. kawachii* and *A. acidus*/*A. foetidus*, which are now both considered subspecies of *A. luchuensis*. Finally, a test group of 42 aegerolysins from 23 different species of *Aspergillus* was established, unless otherwise indicated.

The analysis of the amino-acid conservation between NigA and OlyA sequences was performed with ClustalW (Kyoto University Bioinformatics Centre). The phylogenetic analysis of the aegerolysins in the investigated data set was performed with Molecular Evolutionary Genetics Analysis, version 10.05 (MEGAX) [51,52]. Orthologous were searched in OrthoMCL DB, version 6.1 (beta) [53,54]. Synteny was inferred using Sybil, a web-based software and comparative genomics tool integrated into AspGD [55].

The testing set included genomes from: *A. aculeatus* ATCC 16872 (Aspac1) [21], *A. brasiliensis* CBS 101740 (Aspbr1) [21], *A. campestris* IBT 28561 (Aspcam1) [24], *A. carbonarius* ITEM 5010 (Aspca3) [21], *A. clavatus* NRRL 1 [56], *A.* (ex *Neosartoria*) *fischeri* NRRL 181 (Neofi1) [56], *A. flavus* NRRL3357 (Aspfl1) [56], *A. fumigatus* Af293 (Aspfu1) [57], *A. glaucus* CBS 516.65 (Aspgl1) [21], *A. luchuensis* (ex *kawachii*) IFO 4308 (Aspka1) [58], *A. luchuensis* (ex *A. acidus*/*A. foetidus*) CBS 106.47 (Aspfo1) [21], *A. nidulans* FGSC A4 (Aspnid1) [56], *A. niger* N402, ATCC 1015 [59] or CBS 513.88 (Aspni7) [60], *A. novofumigatus* IBT 16806 (Aspnov1) [24], *A. ochraceoroseus* IBT 24754 (Aspoch1) [24], *A. oryzae* RIB40 (Aspor1) [56], *A. steynii* IBT 23096 (Aspste1) [24], *A. sydowii* CBS 593.65 (Aspsy1) [21], *A. terreus* NIH2624 (Aspte1) [56], *A. tubingensis* CBS 134.48 (Asptu1) [21], *A. versicolor* CBS 583.65 (Aspve1) [21], *A. wentii* DTO 134E9 (Aspwe1) [21], and *A. zonatus* CBS 506.65 (Aspzo1) [21].

The presence of signal sequences, transmembrane domains (TM) and localization was predicted by several different algorithms: EffectorP [61,62], Octopus [63,64], Phobius [65,66], PredGPI [67,68], PrediSi [69,70,71], PredαTM and PredβTM [71,72,73], SecretomeP [74,75], SignalP [76,77], TargetP [78,79], TMHMM [80], and Wolfpsort [81,82]. EffectorP predicts fungal effector proteins in secretomes by machine learning [61,62]. Octopus uses a combination of artificial neural networks and the Hidden Markov model (HMM); the prediction of the transmembrane domain (TM) of proteins and their topology is based on homology searches using the BLAST program [63,64]. Phobius predicts the transmembrane topology and a signal peptide from the amino acid sequence of proteins [65,66]. PredGPI: several eukaryotic proteins associated with the extracellular leaflet of the plasma membrane carry a glycosylphosphatidylinositol (GPI) anchor, which is connected to the C-terminal residue after proteolytic cleavage, which takes place at the so-called ω-site. The prediction of GPI anchored proteins is based on the support vector method for differentiating anchor signals and on the HMM for predicting the most probable ω-site [67,68]. PrediSi predicts the signal peptide and cleavage site in eukaryotes and bacteria via a positional weight matrix approach, improved by a frequency correction that takes into account the amino acid bias present in the proteins in consideration. The software was trained with sequences extracted from the latest recent version of the SwissProt database [69,70]. PredαTM and PredβTM are two independent algorithms that predict α- or β-transmembrane segments of integral membrane proteins by a support vector machine classifier trained on sequence data of transmembrane proteins with known structures. The algorithms predict probable transmembrane regions based on the amino acid adjacency frequency and the position-specific preference of amino acids in the transmembrane regions [71,72,73]. SecretomeP ab initio predicts non-classical protein secretion, i.e., protein secretion not triggered by signal peptides; the method is based on obtaining information about different post-translational and localization proteins involved in the final prediction of secretion [74,75]. SignalP is based on a combination of different neural networks; the method predicts the presence and location of three types of signal peptides and cleavage sites in eukaryotes: secretory signal peptides transported by the Sec-translocon and cleaved by Signal Peptidase I (Sec/SPI); lipoprotein signal peptides transported by the Sec-translocon and cleaved by Signal Peptidase II (Sec/SPII); and Tat signal peptides transported by the Tat-translocon and cleaved by Signal Peptidase I (Tat/SPI) [76,77]. TargetP predicts subcellular localization of eukaryotic proteins based on the presence of any N-terminal sequence, such as signal peptide, mitochondrial transit peptide, chloroplast transit peptide, or thylakoid luminal transit peptide [78,79]. TMHMM predicts transmembrane helixes in proteins [80]. Wolfpsort predicts the subcellular localization of the protein based on the conversion of the protein sequence of amino acids into numerical functions of localization and based on comparison with the amino acid sequences of neighboring organisms [81,82].

## 3. Results

### 3.1. Alignment of Nigerolysins A

**Figure 2:**
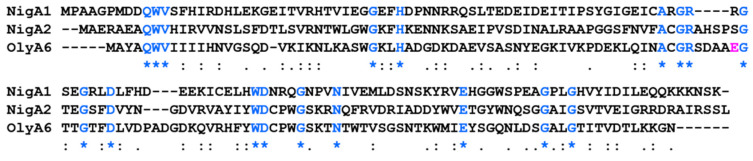
Alignment of nigerolysins A1 and A2, and ostreolysin A6 protein sequences. NigA1 and NigA2, nigerolysin A1 and A2, respectively; OlyA6, ostreolysin A6. In blue, identical amino acid residues (by ClustalW). In magenta, a single glutamic acid residue enables the specific recognition of cholesterol-associated sphingomyelin in OlyA6.

Alignment of NigA and OlyA6 protein sequences shows a low amino acid identity between the aegerolysin pairs (NigA1: NigA2 = 24%; NigA1: OlyA6 = 15% and NigA2: OlyA6 = 35%; Figure 2). The specific recognition of cholesterol-associated sphingomyelin by OlyA6 has been proposed to be driven by a single amino acid residue of glutamic acid (E69) [83], while the recognition site for CPE has not yet been discovered. The corresponding glutamic acid residue in nigerolysins A is replaced by arginine (NigA1) or serine (NigA2), which is consistent with the lipid specificity of NigA2. No binding to cholesterol-associated sphingomyelin was observed, only to cholesterol-associated CPE [34].

### 3.2. Prediction of Localization for Nigerolysins A

The prediction methods used are divided into three groups: those that predict the presence of signal peptides or of TM, or those that predict targeting compartments. The prediction of the secretion of NigA proteins compared to OlyA6 is shown in Table 3.

For the prediction of signal peptides, we applied three different algorithms: SignalP and PrediSi were set to default eukaryotic parameters, and SecretomeP to default mammalian parameters. Prediction with all three methods suggested that these three proteins did not have secretory signal peptides that would allow them to be transported through the Sec translocon and cleaved by SPI, but they were probably non-classically secreted. For OlyA6, the probability/specificity of predicting that the protein would not be classically secreted was somewhat lower (Table 3). The secretion-inferring algorithm SignalP was then used to predict the secretion of aegerolysins in the test set of 23 *Aspergillus* species. The SignalP tool predicted non-conventional protein secretion for all but one of the 42 aegerolysins identified in these species; Aegerolysin Aa-Pri1 precursor (Aspfl1|29773*) exhibits several deviating characteristics. The protein sequences marked with an asterisk (*) were different when obtained from different sources so that a possible erroneous annotation cannot be excluded.

The presence of TM was predicted by four general algorithms: Octopus, TMHMM, PredαTM, and PredβTM. The prediction of all methods for all three tested proteins was almost equivocal, no TM regions were present. The only exception was the chance of some βTM regions in NigA2, but considering that aegerolysins are β-structured proteins, such a prediction was perhaps not particularly surprising if the discrimination fidelity of this method is not optimal (Table 3). The TMHMM tool was then used to predict TM for the test set of 23 *Aspergillus* species; none of the aegerolysins in this group had TM.

The default settings for Phobius and PredGPI were used to determine the putative target compartments; the TargetP tool was set to non-plant parameters; Wolfpsort was set to default parameters for fungi; EffectorP predictions for fungi were applied. The results of localization specificity for the three aegerolysins were mixed and not very uniform; most likely, they were cytosolic and/or extracellular, but not associated with the extracellular leaflet of the plasma membrane by glycosylphosphatidylinositol (Table 3). The fungal Wolfpsort prediction on the test set of 23 *Aspergillus* species suggested that 18 sequences were primarily nuclear, 13 cytoplasmic, 6 cytoplasm/nuclear, 4 extracellular, and 1 mitochondrial. Besides NigA2, the extracellular targeting of the AspHS orthologue in *A. fumigatus* and putative aegerolysin Aa-Pri1 precursor—Aspfl1|29773* from *A. flavus* was proposed. NigA2 and OlyA6 could be regarded as putative fungal effectors; indeed, one third (14) of the 42 proteins investigated were predicted by the EffectorP to be effectors.

### 3.3. Secretion of Nigerolysins A

**Figure 3:**
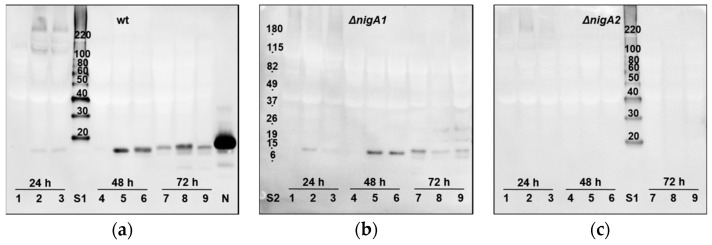
Secretion into the medium of nigerolysins A from *Aspergillus niger*. Detection of nigerolysins in the secretomes during growth and development in the liquid minimal medium with 1% (*w*/*v*) glucose for 24, 48, or 72 h: (**a**) of the wild-type *A. niger* strain N402, (**b**) of *A. niger* mutant strains ∆*nigA1*, and (**c**) ∆*nigA2*. Residual fermented media from each shake flash (<100 mL) were removed from the mycelia and concentrated to 0.5 mL. Secreted proteins were separated by SDS-PAGE gel electrophoresis and transferred to PVDF membrane. Nigerolysins were then detected using Western blotting, with mouse polyclonal antibodies against recombinant NigA2 as primary antibodies [34]. The proteins were detected using the enhanced chemiluminescence method (Pierce ECL Western Blotting substrate; Thermo Fisher Scientific, USA). wt, wild-type (*A. niger* strain N402); ∆*nigA1*, *A. niger* strain N402 with the deleted gene for nigerolysin NigA1; ∆*nigA2*, *A. niger* strain N402 with the deleted gene for nigerolysin NigA2. Independently grown triplicates for each time point: 1–3, 24 h; 4–6, 48 h; 7–9, 72 h; N, recombinant nigerolysin NigA2 [34]; S1, MagicMarkTM XP Western Protein Standard (kDa; Thermo Fisher Scientific, USA); S2, BenchMarkTM Pre-Stained Protein Standard (kDa; Thermo Fisher Scientific, USA); calculated MW sizes of aegerolysins NigA1 and NigA2 are almost the same, 16.256 and 16.259 kDa, respectively.

The secretion of nigerolysins A from *A. niger* colonies grown on the agar plates was previously confirmed with NigA2-specific antibody [34]. Here, we have further elaborated on the immunodetection of the secretion of nigerolysins A into the liquid medium. Nigerolysins A were detected in the secretomes in the liquid medium during one to three days of growth in shaking flasks. As expected, nigerolysin NigA2 was detected at the predicted size in the wild-type *A. niger* strain N402 and the *A. niger* ∆*nigA1* mutant strain, but not in the ∆*nigA2* mutant strain (Figure 3a–c, respectively).

### 3.4. Immunocytochemistry and Live Cell Imaging of Nigerolysin A Proteins in Hyphae

**Figure 4:**
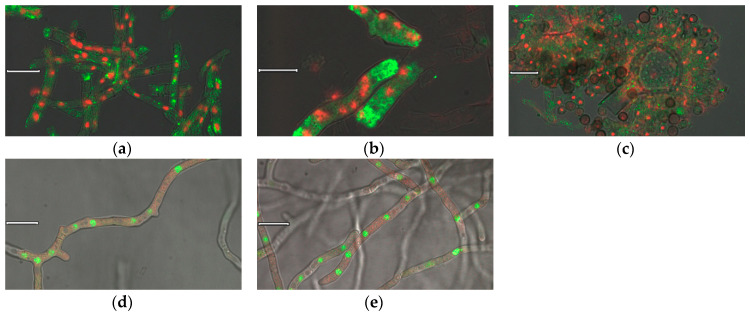
Cell imaging of NigA proteins. Immunolocalization of NigA proteins in the fungus *Aspergillus niger* wild-type strain: NigA2 protein localized by polyclonal anti-NigA2 antibody and fluorescent labeled secondary antibody (in green), and nuclei stained by DAPI (in red): (**a**) Fungal hyphae; (**b**) Hyphal tip; (**c**) Conidiophore. Live cell imaging of NigA proteins in the *A. niger* NigA-FP mutant strains, nuclei stained by Sitox Green (in green): (**d**) NigA1-mCherry protein (in red), and (**e**) NigA2 protein-mCherry protein (in red); Size of a ruler line was 10 μm.

Furthermore, antibodies were used for the immunocytochemical detection of nigerolysins A in the fixed cryoresins of *A. niger* wild-type hyphae. After binding specific antibodies to NigA2 proteins, these were detected by fluorescent-labeled secondary antibodies, which are shown in green in Figure 4a–c. The cell nuclei shown in red were labeled with nucleic acid dye. NigA2 proteins appeared to be localized in the cytoplasm of fungal hyphae and not only in the hyphal tips where the classical protein secretion normally takes place. Immunostained NigA2 protein was also observed in conidiophores, but it was less pronounced in non-germinating conidia with thick cell walls, although nuclei were also stained there (Figure 4c). Due to the technique used, the hyphae were more fragmented.

We investigated the same localization with another technique, the live-cell imaging of NigA proteins in the *A. niger* transformants. Here, fusions of nigerolysin A with fluorescent protein, NigA1-mCherry and NigA2-mCherry, were marked red, and nuclei stained with nucleic acid-binding vital dye were marked green (Figure 4d,e, respectively). Both NigA proteins seemed to be similarly distributed in the fungal hyphae, evenly distributed throughout the cytoplasm.

### 3.5. Live Cell Colocalization of Nigerolysin A Proteins in Hyphae

**Figure 5:**
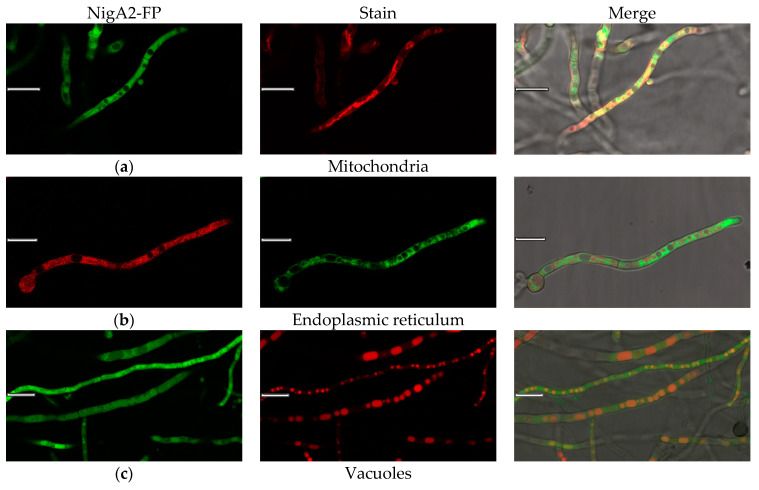

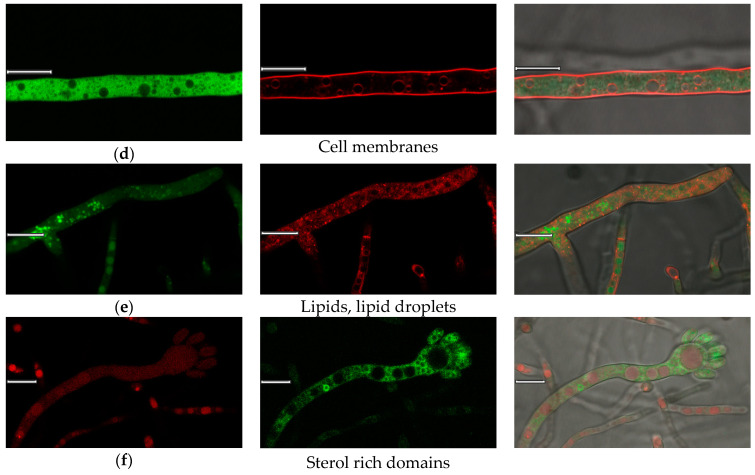
Colocalization of NigA2-fluorescent fusion protein and vital dyes in the fungus *Aspergillus niger*. NigA2-eYFP (in green) or NigA2-mCherry fusion-proteins (in red) are merged with dyes: (**a**) Mito Tracker® Deep Red; (**b**) ER-Tracker™ Blue-White DPX; (**c**) Cell Tracker™ CMAC Blue; (**d**) Synapto Red™; (**e**) Nile Red; and (**f**) Filipin. Size of a ruler line was 10 μm.

To further elaborate the compartmentalization observed by immunolocalization of native NigA proteins and fusion proteins NigA-FP in *A. niger* hyphae, we performed colocalization studies by live-cell imaging and vital staining (Figure 5a–f). Vital dyes have limited compatibility with fluorescent fusion proteins, so more combinations were required to cover different cell compartments. NigA proteins did not appear to colocalize with cell structures such as mitochondria or endoplasmic reticulum but fluorescent signals were occasionally found in vacuoles (Figure 5a–c). NigA proteins did not bind to their own cell membranes, lipids, lipid droplets, or sterol rich domains (Figure 5d–f). In general, fluorescent protein signals dilute and decrease in slightly acidic extracellular environments.

### 3.6. Chromosomal Loci of Aegerolysins Genes

Further bioinformatics investigations were carried out to ascribe the similarity of the aegerolysins in the test group with the intention to infer their possible function. 

**Figure 6:**
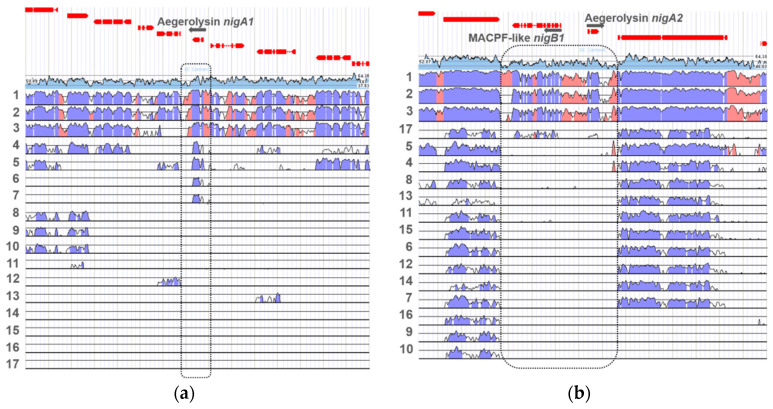
Loci of aegerolysin genes in *Aspergillus niger* CBS 513.88. Nucleotide conservation for the aegerolysin *nigA1* (supercontig 19:43975-59064) (**a**), and *nigA2* gene loci (supercontig 01:2367156-2382065) (**b**). Loci were compared to genomes of *A. luchuensis* (1), *A. tubingensis* (2), *A. brasilliensis* (3), *A. aculeatus* (4), *A. carbonarius* (5), *A. oryzae* (6), *A. flavus* (7), *A. wentii* (8), *A. sydowii* (9), *A. versicolor* (10), *A. clavatus* (11), *A. glaucus* (12), *A. zonatus* (13), *A. fischeri* (14), *A. terreus* (15), *A. nidulans* (16), and *A. fumigatus* (17). Light blue, GC content; regions of high conservation: dark blue, exons; pink, non-coding. The dotted line indicates (expected) position of aegerolysin loci. Data were collected from JGI MycoCosm [23].

For the first nigerolysin gene locus *nigA1*, different levels of nucleotide conservation were observed compared to other *Aspergillus* species (Figure 6a). Nucleotide conservation was considerably high in the genomes of *A. luchuensis* (ex *kawachii*), *A. tubingensis,* and *A. brasilliensis*, while conservation was reduced in the locus for genomes of *A. aculeatus* and *A. carbonarius*. In *A. flavus* and *A. oryzae*, some nucleotide conservation was observed only in the aegerolysin genes themselves. No nucleotide conservation was observed in the genomes of *A. wentii*, *A. sydowii*, *A. versicolor*, *A. clavatus*, *A. glaucus*, *A. zonatus*, *A. fischeri*, *A. terreus*, *A. nidulans,* and *A. fumigatus*. 

In the other genome locus, the second nigerolysin gene *nigA2* formed a bidirectional gene pair with the MACPF-like gene *nigB1*, which is positioned on the complementary DNA chain (Figure 6b), as did the aegerolysin genes in the genome of *P. ostreatus* (*olyA*/*plyA*) and *plyB*) [34]. At this genome locus, the nucleotide conservation of these two genes was high compared to the genomes of *A. luchuensis* (ex *kawachii*), *A. tubingensis,* and *A. brasiliensis*, and moderate compared to the (subtelomeric) aegerolysin aspHS of *A. fumigatus*. In none of the other genomes of the compared species (i.e., *A. carbonarius*, *A. aculeatus*, *A. wentii*, *A. zonatus*, *A. clavatus*, *A. terreus*, *A. oryzae*, *A. glaucus*, *A. fischeri,* and *A. flavus*) there was no conservation for either aegerolysin or MACPF-like genes, but there was at a reasonable extent on both sites outside these two genes, suggesting the absence of both genes. In *A. nidulans*, *A. sydowii,* and *A. versicolor* these conserved areas were only on the side of the MACPF-like genes. Several MACPF-like proteins may be present in each fungal species, not only those paired with aegerolysins [84]. In *A. niger*, the two genes that encode MACPF-like proteins are called nigerolysins B1 and B2: *nigB1* (An01g09970/ Aspni7|1182338) and *nigB2* (An09g03750/ Aspni7|162837) [34]. Nigerolysin B2 *nigB2* was located elsewhere, on chromosome I.

**Figure 7:**
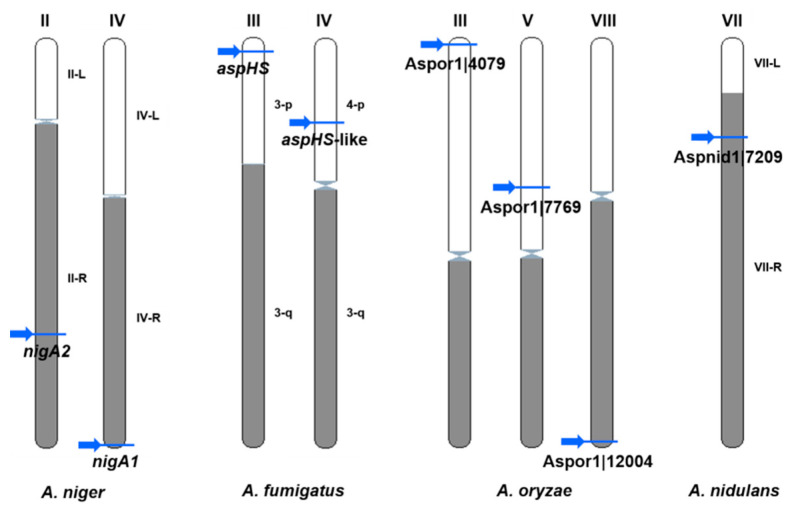
Chromosomal loci of aegerolysins in *Aspergillus* species with known karyotype. *Aspergillus niger* CBS 513.88 chromosome IV: *nigA1*, and chromosome II: *nigA2*; *A. fumigatus* chromosome III: *aspHS* and chromosome IV, *aspHS-like*; *A. oryzae*: chromosome III, Aspor1|4079, chromosome V, Aspor1|7769, and chromosome VII: Aspor1|12004; and *A. nidulans* chromosome VIII: Aspnid1|7209. Arrows, position of aegerolysin genes on chromosomes. Data were obtained from Ensembl Fungi (EMBL-EBI) [47] and CADRE [48].

Although the number of known fungal genome sequences has passed over 1700, only a few species have a complete karyotype. New sequencing techniques involving PacBio and MiniIon, which yield large DNA contigs, will soon lead to a rapid progression of karyotyping. Therefore, it was possible to determine the chromosomal localizations for only eight aegerolysin genes in four species (*A. niger*, *A. fumigatus*, *A. oryzae,* and *A. nidulans*) (Figure 7). 

For example, in one study of *A. fumigatus*, genes that were involved in pathogenicity occurred in pathogenicity islands located near the chromosome the ends in subtelomeric regions [85]. Indeed, half of these (few) genes were located near the telomeres: *nigA1* on the *A. niger* chromosome IV; *aspHS* on the *A. fumigatus* chromosome III; Aspor1|4079 on the *A. oryzae* chromosome III; and Aspor1|12004 on the chromosome VIII. The chromosome overview showed no obvious correlations in terms of density of protein-coding genes, short non-coding genes or pseudogenes, or percentage of GC repeats, except that the *aspHS-like* gene on the *A. fumigatus* chromosome IV was close to a large gap without coding genes (Ensembl Fungi Chromosome summary data not shown).

### 3.7. Aegerolysin Orthologues in Aspergillus Species

**Figure 8:**
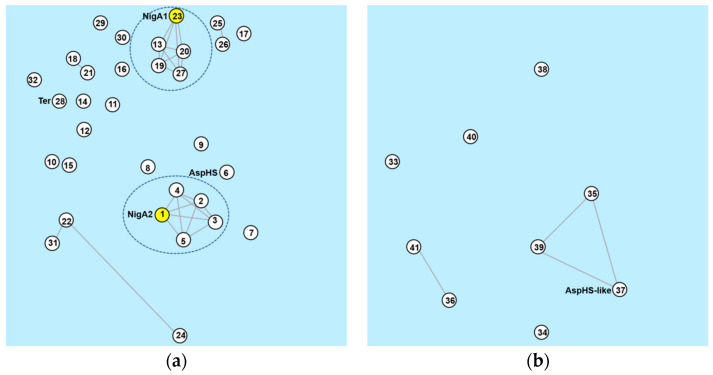
Orthologue group of aegerolysin protein sequences in genus *Aspergillus*. Group OG6_153003 (**a**), and OG6_454485 (**b**). *A. niger* Aspni7|1162650 (1), *A. tubingensis* Asptu1|141875 (2), *A. luchuensis* Aspfo1|39400 (3), *A. brasiliensis* Aspbr1|39684 (4), *A. luchuensis* (ex *kawachii*) Aspka1|172285 (5), *A. fumigatus* Aspfu1|3326 (6), *A. luchuensis* Aspfo1|201212 (7), *A. wentii* Aspwe1|166867 (8), *A. novofumigatus* Aspnov1|380431 (9), *A. oryzae* Aspor1|4079 (10), *A. ochraceoroseus* Aspoch1|502518 (11), *A. campestris* Aspcam1|288126 (12), *A. luchuensis* Aspfo1|63745 (13), *A. glaucus* Aspgl1|171679 (14), *A. sydowii* Aspsy1|47201 (15), *A. carbonarius* Aspca3|518161 (16), *A. aculeatus* Aspac1|40672 (17), *A. versicolor* Aspve1|437845 (18), *A. luchuensis* (ex *kawachii*) Aspka1|183089 (19), *A. tubingensis* Asptu1|60608 (20), *A. sydowii* Aspsy1|41775 (21), *A. sydowii* Aspsy1|41379 (22), *A. niger* Aspni7|1145225 (23), *A. flavus* Aspfl1|29773 (24), *A. oryzae* Aspor1|12004 (25), *A. flavus* Aspfl1|31827 (26), *A. brasiliensis* Aspbr1|197000 (27), *A. terreus* Aspte1|7119 (29), *A. steynii* Aspste1|437249 (29), *A. zonatus* Aspzo1|137411 (30), *A. flavus* Aspfl1|8431* (31), *A. nidulans* Aspnid1|7209 (32), *A. campestris* Aspcam1|323002 (33), *A. clavatus* Aspcl1|4413 (34), *A. fischeri* Neofi1|5318 (35), *A. flavus* Aspfl1|32504* (36), *A. fumigatus* Aspfu1|4834 (37), *A. glaucus* Aspgl1|171679 (38), *A. novofumigatus* Aspnov|409737 (39), *A. ochraceoroseu* Aspoch1|556958 (40), and *A. oryzae* Aspor1|4079 (41); *, sequence from different resources may vary. Each edge is a Blast score between two protein sequences (above E-value threshold 1 × 10^−70^). Data were obtained from OrthoMCL DB [53,54].

Orthologous groups of aegerolysin protein sequences were identified by OrthoMCL DB, not all of them belonged to the same group [53,54]. In the first group OG6_153003, there was a majority of aegerolysins (32 of 42): one of *A. aculeatus*, *A. campestris*, *A. carbonarius*, *A. glaucus*, *A. fumigatus*, *A. nidulans*, *A. novofumigatus*, *A. ochraceoroseus*, *A. steynii*, *A. terreus*, *A. versicolor*, *A. wentii,* and *A. zonatus*; two of *A. brasilliensis*, *A. luchuensis* (ex *kawachii*), *A. niger*, *A. oryzae,* and *A. tubingensis*; three of *A. luchuensis*, *A. flavus,* and *A. sydowii*. Figure 8a shows the group OG6_153003, when the expected cut-off value (E-value) was set to a higher significance (lower than 1 × 10^−70^). Two pentagrams, each clustered around NigA1 or NigA2, emerged from the same group of species: *A. tubingensis*, *A. brasilensis*, *A. luchuensis,* and *A. luchuensis* (ex *kawachii*)). In addition to two associated pairs of aegerolysins: *A. versicolor* Aspve1|437845 paired with *A. sydowii* (Aspsy1|41775), and *A. oryzae* Aspor1|12004 with *A. flavus* Aspfl1|31827, and a triplet, Aspsy1|41379 of *A. sydowii* was joined with Aspfl1|8431* and Aspfl1|29773 of *A. flavus*. In the second group OG6_454485, there were another nine aegerolysins, one from several species: *A. campestris*, *A. clavatus*, *A. fischeri*, *A. flavus*, *A. fumigatus*, *A. glaucus*, *A. novofumigatus*, *A. ochraceoroseus,* and *A. oryzae* (Figure 8b). The Blast score below 1 × 10^−70^ identified in this group identified an additional triangle of aegerolysins (AspHS-like, Neofi1|5318, and Aspnov|409737) and another pair of them from *A. oryzae* Aspor1|4079 and *A. flavus* Aspfl1|32504*. Only the aegerolysin Aspzo1|137037 from *A. zonatus* belonged to the third orthologous group (OG6_170879).

### 3.8. Aegerolysin Synteny among Aspergillus Species

**Figure 9:**
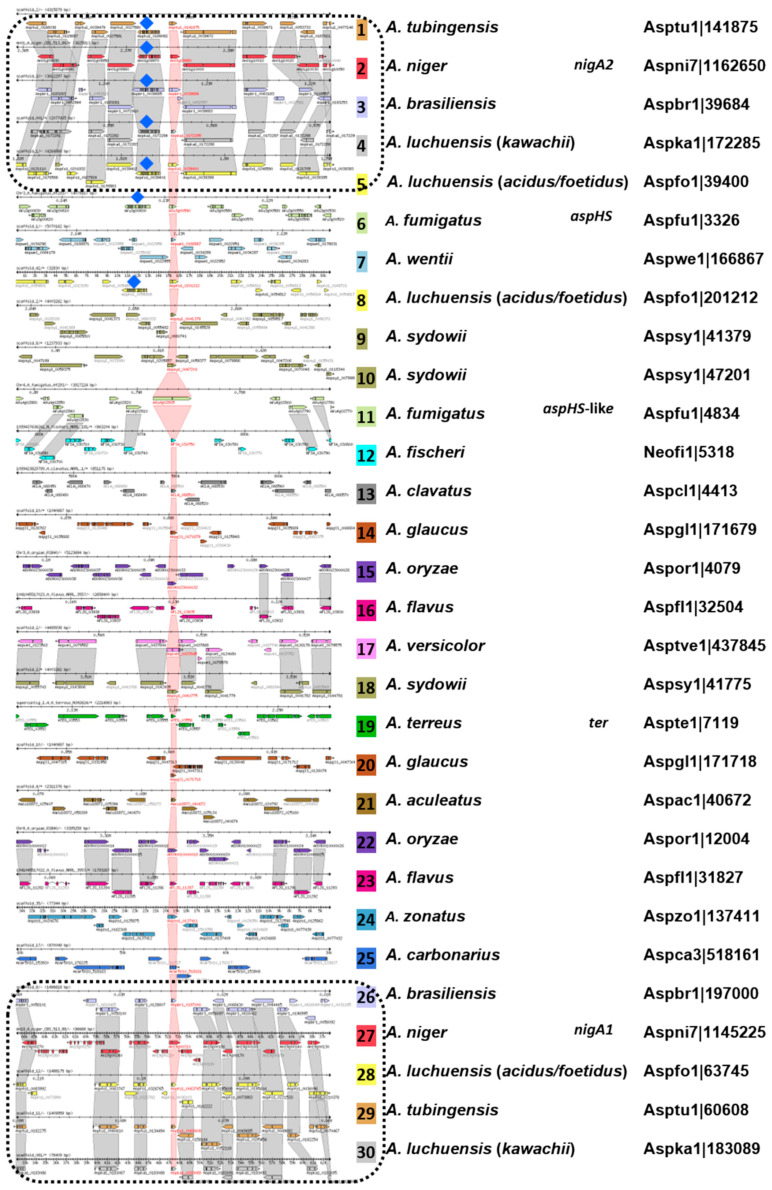
Synteny overview of aegerolysins in *Aspergillus* species. Sybil graphical display of 30 orthologous and paralogous aegerolysin genes from 19 *Aspergillus* species. The aegerolysin genes were centered and highlighted by shading in red. Other syntenic genes in the displayed genomic region were highlighted in grey. Putative MACPF-like proteins were marked by blue diamonds. Sybil data were collected from the *Aspergillus* Genome Database (AspGD) [55,56].

Orthologous and paralogous aegerolysin genes of 19 *Aspergillus* species were clustered with the comparative genomics tool Sybil in AspGD [46,55], (species not included in this analysis: *A. campestris*, *A. novofumigatus*, *A. ochraceoroseus,* and *A. steynii*). In general, there were three groups of aegerolysin genes. In addition to the more or less non-syntenic ones, there were two syntenic blocks, including *nigA1* or *nigA2* (represented by grey hatched regions in Figure 9), which were composed of four closely related species (*A. tubingensis*, *A. brasillensis*, *A. luchuensis,* and *A. luchuensis* (ex *kawachii*)). Also, in *A. versicolor* and *A. sydowii* a certain synteny of neighboring genes was observed, in *A. fumigatus* and *A. fischeri* or *A. oryzae* and *A. flavus* only partially.

The identification and annotation of putative MACPF-like proteins are more prone to error due to the higher intron numbers in these genes. In the syntenic block containing *nigA2*, this aegerolysin gene was paired with the MACPF-like gene *nig*B1; the same was true also for the other four species from this block: *A. tubingensis*, *A. brasillensis*, *A. luchuensis,* and *A. luchuensis* (ex *kawachii*)). Putative MACPF-like genes could also be paired with *aspHS* from *A. fumigatus*, another aegerolysin from *A. luchuensis*, and perhaps also from *A. wentii* (marked by blue diamonds in Figure 9).

### 3.9. Aegerolysin Phylogeny of the Aspergillus Species

A phylogenetic relationship for the *Aspergillus* species ordered within the *Eurotiales*, family *Aspergillaceae*, genus *Aspergillus* was recently described by Houbraken et al. [22]. The set of species studied was taxonomically well distributed, as follows: subgenus *Circumdati* (1): section *Nigri*: *A. tubingensis*, *A. luchuensis*, *A. niger*, *A. brasillensis*, *A. carbonarious,* and *A. aculeatus*; section *Terrei*: *A. terreus*; section *Circumdati*: *A. campestris* and *A. steynii*; section *Flavi*: *A. flavus* and *A. oryzae*. Subgenus *Nidulantes* (2), section *Nidulantes*: *A. nidulans*, *A. sydowii,* and *A. versicolor*; section *Ochraceorosei*: *A. ochraceoroseus*. Subgenus *Fumigati* (3), section *Fumigati*: *A. fumigatus*, *A. fischeri,* and *A. novofumigatus*; section *Clavati*: *A. clavatus*. Subgenus *Aspergillus* (4), section *Aspergillus*: *A. glaucus*. Subgenus *Cremei* (5), section *Cremei*: *A. wentii*. The species *A. zonatus* is no longer listed as *Aspergillus* anymore but as *Penicilliopsis zonata*.

**Figure 10:**
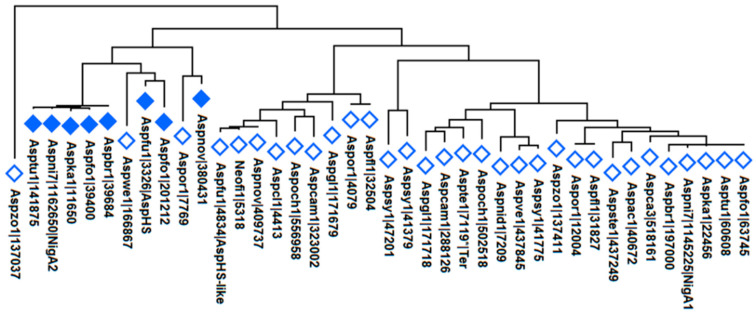
Phylogenetic analysis of aegerolysins in *Aspergillus* species. Evolutionary analysis including 40 aegerolysin protein sequences was inferred using MEGAX [52], Aspfl1|27858* and Aspfl1|29773* were omitted due to conflicting annotation data. Molecular phylogenetic analysis was performed using the maximum likelihood method and JTT matrix-based model [51] after ClustalW alignment. All positions containing gaps and missing data were eliminated. There were 210 positions in the final dataset. *, sequence from different resources may vary; closed diamonds, aegerolysin with syntenic MACPF-like proteins.

At least one to four aegerolysins have been found per species: one in *A. aculeatus*, *A. carbonarius*, *A. nidulans*, *A. steynii*, *A. terreus*, *A. versicolor*, *A. wentii*, *A. zonatus*, and in *A. fischeri*; two in *A. brasillensis*, *A. campestris*, *A. fumigatus*, *A. luchuensis* (ex *kawachii*), *A. niger*, *A. novofumigatus*, *A. ochraceoroseus* and *A. tubingensis*, three in *A. luchuensis*, *A. oryzae* and *A. sydowii*, and four in *A. flavus*; a total 42 aegerolysins in 23 species of *Aspergillus*. The phylogenetic analysis of these aegerolysins is shown in Figure 10. 

## 4. Discussion

### 4.1. Non-Conventional Protein Secretion (i.e., Not Signal Peptide Triggered Secretion)

Filamentous fungi show an enormous nutritional flexibility as well as metabolic and secretion capacity. The conventional secretory pathway (exocytosis) delivers cargo from the endoplasmic reticulum through the *trans*-Golgi network to the plasma membrane at the hyphal tips, subapical regions, or septa. However, such signal peptide dependent protein secretion cannot successfully explain the increasing number of proteins without signal peptides that are located outside the plasma membrane, such as nigerolysins A. 

The process by which such leaderless secretory proteins gain access to the outside of the cell is called unconventional protein secretion (UPS). UPS was investigated in some yeast cells and fungi [86,87]. UPS may be associated with vesicles, e.g., some secretory proteins reach the plasma membrane without passing through the Golgi apparatus (i), or through multi-vesicular bodies fused to the plasma membrane (ii). Autophagy, as a conserved degradation process of eukaryotes, which mediates the incorporation of cytoplasmic proteins and organelles into lysosomes/vacuoles for bulk degradation, enabling the recycling of cytoplasmic components as nutrients to support cell survival, could also be one of the mechanisms of UPS (iii). Another mechanism of UPS could be a potential lock-type that allows chitinase to be actively translocated into the fragmentation zone connecting dividing mother and daughter cells, where it supports cell division by breaking down residual chitin (iv) [88]. For nigerolysins, we propose an additional pathway for UPS by simply releasing the cell content from broken cells created by feeding predators. A high abundance of these small but very robust proteins after 48 and even 72 h (Figure 3a) could indicate an additional release by autolysis processes next to broken hyphal cells due to fungal growth in shaking cultures.

### 4.2. Application of Aegerolysin Secretion Pathway in the Production of Non-Fungal Proteins

The major advantages of filamentous fungi as protein producers include high yields and efficient secretion of their bioactive proteins with post-translational modifications, affordable growth media, and relatively simple genetic manipulation. On the other hand, a considerable number of recombinant non-fungal proteins are lost in the secretion pathway due to incorrect post-translational processing [89]. Several approaches with limited efficacy have already been proposed to overcome the potential productivity limitations caused by secretion bottlenecks. Aegerolysin promoters have been reported to efficiently overexpress several homologous and heterologous genes [8,10]. It would be ideal to combine these promoters with the uncovered mechanism of aegerolysin secretion to achieve a more efficient secretion of non-fungal recombinant proteins, but the proposed route of aegerolysins does not seem to be suitable for this purpose.

### 4.3. Function of Aergerolysins

Bando et al. [8] reported a strong promoter from a hemolysin-like gene (*hlyA*), which codes for an aegerolysin-like protein. This *hlyA* promoter is not suppressed by glucose and is activated by conditions or factors that are not limited to sporulation. In fact, besides the most common *cis* elements in the promoter regions of numerous eukaryotic genes, several heat shock transcription factor binding sequences and *BrlA* binding sequences were identified to affect the transcription [8]. Heat shock transcription factors positively regulate the stress response gene family, and the *brlA* gene is a regulator of conidiation in *A. oryzae*. 

Such a strong promoter could influence the cytoplasmic expression of nigerolysins A in entire hyphae, which was observed in our experiments. The stress response in the presence of predatory invertebrates could further enhance the synthesis of these proteins at the site of feeding. In fact, it has been already reported, that Aspergilli are rejected as food by several species of invertebrate decomposers, and secretion of nigerolysins might be one of the reasons for the observed effect [90].

Phagocytosis by predators, which leads to broken cells, thus could enable the release of even more nigerolysins A (aegerolysins), which bind to the predator membrane lipids. Such CPE-bound nigerolysins could enable further binding of partner MACPF-like proteins (nigerolysins B) to support the likely pore formation in the lipid membrane as a defense mechanism against predatory invertebrates. In general, MACPF-like proteins in fungi most likely contribute to various specific processes [84], while some MACPF-like proteins are intracellular, others are found in secretomes (also without a typical SP) [32], and some of them may be involved in the pathogenesis.

Aegerolysin orthologues found in the syntenic blocks containing *nigA2* paired with *nigB1* gene code for proteins most likely have the same or similar function described for the pesticide proteins pairs OlyA6/PlyB, PlyA2/PlyB, and EryA/PlyB from oyster mushrooms (*Pleurotu*s sp.). The phylogenetic relationship further supports this assumption. In the genus *Aspergillus*, subgenus (1) *Circumdati*, section *Nigri*; the series *Nigri* comprises five species *A. tubingensis*, *A. luchuensis*, *A. luchuensis* (ex *kawachii*), *A. niger,* and *A. brasillensis* [22] having MACPF-like genes paired to aegerolysins. Genes for AspHS from *A. fumigatus* and aegerolysin orthologs from *A. luchuensis*, *A. novofumigatus,* and perhaps also from *A. wentii* also appear to be paired with MACPF-like genes. It is more difficult to expect that the same function is shared for all aegerolysins. For example, the recently functionally characterized stand-alone AGL1 aegerolysin in the mycoparasitic fungus *Trichoderma atroviride* reveals a role in conidiation and antagonism [91].

## 5. Conclusions

We have experimentally confirmed the secretion of nigerolysins A from the fungus *A. niger*, which was cultivated in shaking flasks for up to three days. Immunocytochemistry and live-cell imaging of NigA proteins showed that these proteins were localized in the cytoplasm and not only in the hyphal tips where classical protein secretion normally occurs. NigA proteins did not bind to their own cell membranes, lipid droplets or sterol-rich domains, or colocalize within cell structures such as mitochondria or endoplasmic reticulum, except occasionally in vacuoles.

To broaden the view to the level of the genus *Aspergillus*, a number of taxonomically well distributed 23 species were also assessed by bioinformatics. In these species, we identified at least one to four aegerolysin sequences, which together yield 42 aegerolysins. Due to the absence of SP and TM, the prediction tools inferred that the protein secretion was non-conventional. One-third of these proteins were predicted to be effectors, small secreted proteins that are species- or even lineage-specific and alter the structure, metabolism, and function of the host cell. 

When comparing the two loci of the *nigA* gene of *A. niger* with other *Aspergillus* species, different levels of nucleotide conservation were observed. Half of these genes, from the few complete genomes to the chromosomes, were located near the telomeres, as is assumed for genes in pathogenicity islands. In addition to the more or less non-syntenic regions, two highly syntenic regions with *nigA1* or *nigA2* appeared among the *Aspergillus* genomes, both consisting of four closely related species.

Finally, in accordance with the phylogeny for the *Aspergillus* species (subgenus *Circumdati*, section/series *Nigri*), we propose aegerolysin orthologues found in the syntenic blocks consisting of aegerolysin paired with MACPF-like gene code for proteins that most likely have the same or similar function as the pesticide–protein pairs from oyster mushrooms (*Pleurotus* sp.), which are directed against the CPE lipid target. We also propose an additional UPS pathway for aegerolysins by the simple release of cells content from broken cells resulting from feeding invertebrates, or by autolysis processes due to broken hyphal cells.

## Figures and Tables

**Table 1:** List of primers used for this paper.

Primers	Sequence (5′→3′)
*nigA1Not*IF	agtaca**ggcggccgc**atgcctgcagccggcc
*nigA1Eco*RIR	gctc**gaattc**tttgctattcttcttcttctgctgc
*nigA2Not*IF	agtaca**ggcggccgc**atggccgagcgagcagaag
*nigA2Eco*RIR	gctc**gaattc**aagacttgaccgaatcgcacg

Underlined and bold nucleotides in sequences, introduced restriction sites of enzymes *Not*I and *Eco*RI; F, forward primer; R, reverse primer; *nigA1*, nigerolysin A1 gene; and *nigA2*, nigerolysin A2 gene.

**Table 2:** Properties of applied dyes and fluorescent proteins.

Dye/Fluorescent Protein	Selectivity	Working Concentration	Incubation Time	Absorption/Emission (nm)
Alexa Fluor 488 Dye	NigA2 antibody	n. a.	n. a.	495/519
Cell Tracker™ CMAC Blue (Molecular probes C2110)	Vacuole	0.5–5 µM	30 min	353/466
DAPI (Termo Fisher 62248)	Nucleus, nucleic acids	0.3 µM	5 min	360/460
eYFP	NigA2-FP protein	n. a.	n. a.	514/520–570
ER-Tracker™ Blue-White DPX (Invitrogen E12353)	Endoplasmic reticulum	0.1 µM–1 µM	20 min	374/430–640
Filipin (Sigma F9765)	Sterol rich domains	0.05 mg/mL	15 min	340–380/385–470
mCherry	NigA2-FP protein	n. a.	n. a.	514/587–610
Mito Tracker® Deep Red (Molecular probes M22426)	Mitochondria	0.1 µM–0.5 µM	15–45 min	644/665
Nile Red (Invitrogen N1142)	Lipids, lipid droplets	1–10 µg/mL	15–30 min	552/636
Synapto Red™ (Biotium 70027)	Cell membranes	0.02 µM–0.2 µM	15–45 min	510/750
Sytox Green (Invitrogen S7020)	Nucleus, nucleic acids	0.01 µM–1 µM	10 min	504/523

FP, fluorescent protein; n. a., not applicable.

**Table 3:** Sequence analysis of aegerolysin proteins by web prediction tools.

Tool	NigA1	Probability/Specificity	NigA2	Probability/Specificity	OlyA6	Probability/Specificity
SignalP-5.0 (Eukarya) [76,77]	Sec/SPI Other SP	0.0006 0.9994	Sec/SPI Other SP	0.0008 0.9992	Sec/SPI Other SP	0.0129 0.9871
PrediSi (Eukaryotic) [69,70]	Cleavage position: 37; not predicted for secretion	0	Cleavage position: 33; not predicted for secretion	0.043	Cleavage position: 16; not predicted for secretion	0.1810
SecretomeP 2.0 (Mammalia) [74,75]	Non-classically secreted 0.688	2.126	Non-classically secreted 0.875	4.910	Non-classically secreted 0.329	0.616
Octopus [63,64]	No TM-regions; in a globular region, further than 23Å from the membrane	/	No TM-regions; in a globular region, further than 23Å from the membrane	/	No TM-regions; in a globular region, further than 23Å from the membrane	/
TMHMM-2.0 [80]	No membrane helices	/	No membrane helices	/	No membrane helices	/
PredαTM [71,72]	Not probable		Not probable		Not probable	
PredβTM [71,73]	Probable	20–32	Probable	14–20; 27–36; 59–68; 86–97; 128–137	Not probable	-
Phobius [65,66]	Non-cytoplasmic	/	Non-cytoplasmic	/	Non-cytoplasmic	/
PredGPI [67,68]	Not GPI-anchored; ω-position: 114	24.4%	Not GPI-anchored; ω-position: 123	84.6%	Not GPI-anchored; ω-position: 114	16.0%
TargetP-2.0 (Non-plant) [78,79]	Other SP Mitochondrial transfer peptide	0.9999 0 0.0001	Other SP Mitochondrial transfer peptide	0.9947 0.0022 0.0032	Other SP Mitochondrial transfer peptide	0.7305 0.2678 0.0017
Wolfpsort (Fungi) [81,82]	Nucleus Cytosol/nucleus Cytosol Peroxisome Mitochondria Extracellular Vacuole	12.5 11 8.5 3 1 1 1	Extracellular Cytosol Cytosol/nucleus Cytosol/mitochondria Nucleus Mitochondria	14 8.5 6.833 5.666 3 1.5	Extracellular Cytosol Cytosol/nucleus Mitochondria Vacuole Nucleus	12 9.5 6 2 2 1.5
EffectorP 2.0 [61,62]	Non-effector	0.701	Effector	0.731	Effector	0.577

NigA1 and NigA2, nigerolysin A1 and A2, respectively; OlyA6, ostreolysin A6; SP, signal peptide; TM, transmembrane domain; HMM, hidden Markov model; GPI, glycosylphosphatidylinositol; Sec/SPI, Sec translocon and cleaved by signal peptidase I.

## References

[1] Berne S., Lah L., Sepčić K. (2009). Aegerolysins: Structure, function, and putative biological role. Protein Sci..

[2] Novak M., Kraševec N., Skočaj M., Maček P., Anderluh G., Sepčić K. (2015). Fungal aegerolysin-like proteins: Distribution, activities, and applications. Appl. Microbiol. Biotechnol..

[3] Butala M., Novak M., Kraševec N., Skočaj M., Veranič P., Maček P., Sepčić K. (2017). Aegerolysins: Lipid-binding proteins with versatile functions. Semin. Cell Dev. Biol..

[4] Sepčić K., Berne S., Rebolj K., Batista U., Plemenitaš A., Šentjurc M., Maček P. (2004). Ostreolysin, a pore-forming protein from the oyster mushroom, interacts specifically with membrane cholesterol-rich lipid domains. FEBS Lett..

[5] Ota K., Leonardi A., Mikelj M., Skočaj M., Wohlschlager T., Künzler M., Aebi M., Narat M., Križaj I., Anderluh G. (2013). Membrane cholesterol and sphingomyelin, and ostreolysin A are obligatory for pore-formation by a MACPF/CDC-like pore-forming protein, pleurotolysin B. Biochimie.

[6] Skočaj M., Resnik N., Grundner M., Ota K., Rojko N., Hodnik V., Anderluh G., Sobota A., Maček P., Veranič P. (2014). Tracking cholesterol/sphingomyelin-rich membrane domains with the ostreolysin A-mCherry protein. PLoS ONE.

[7] Bhat H.B., Ishitsuka R., Inaba T., Murate M., Abe M., Makino A., Kohyama-Koganeya A., Nagao K., Kurahashi A., Kishimoto T. (2015). Evaluation of aegerolysins as novel tools to detect and visualize ceramide phosphoethanolamine, a major sphingolipid in invertebrates. FASEB J..

[8] Bando H., Hisada H., Ishida H., Hata Y., Katakura Y., Kondo A. (2011). Isolation of a novel promoter for efficient protein expression by *Aspergillus oryzae* in solid-state culture. Appl. Microbiol. Biotechnol..

[9] Hisada H., Tsutsumi H., Ishida H., Hata Y. (2013). High production of llama variable heavy-chain antibody fragment (VHH) fused to various reader proteins by *Aspergillus oryzae*. Appl. Microbiol. Biotechnol..

[10] Hatzmann M. (2011). Constitutive Promoter Application. European Patent.

[11] Tomita T., Noguchi K., Mimuro H., Ukaji F., Ito K., Sugawara-Tomita N., Hashimoto Y. (2004). Pleurotolysin, a Novel Sphingomyelin-specific Two-component Cytolysin from the Edible Mushroom *Pleurotus ostreatus*, Assembles into a Transmembrane Pore Complex. J. Biol. Chem..

[12] Bhat H.B., Kishimoto T., Abe M., Makino A., Inaba T., Murate M., Dohmae N., Kurahashi A., Nishibori K., Fujimori F. (2013). Binding of a pleurotolysin ortholog from *Pleurotus eryngii* to sphingomyelin and cholesterol-rich membrane domains. J. Lipid Res..

[13] Shibata T., Kudou M., Hoshi Y., Kudo A., Nanashima N., Miyairi K. (2010). Isolation and characterization of a novel two-component hemolysin, erylysin A and B, from an edible mushroom, *Pleurotus eryngii*. Toxicon.

[14] Lukoyanova N., Kondos S.C., Farabella I., Law R.H.P., Reboul C.F., Caradoc-Davies T.T., Spicer B.A., Kleifeld O., Traore D.A.K., Ekkel S.M. (2015). Conformational Changes during Pore Formation by the Perforin-Related Protein Pleurotolysin. PLoS Biol..

[15] Resnik N., Repnik U., Kreft M.E., Sepčić K., Maček P., Turk B., Veranič P. (2015). Highly Selective Anti-Cancer Activity of Cholesterol-Interacting Agents Methyl-β-Cyclodextrin and Ostreolysin A/Pleurotolysin B Protein Complex on Urothelial Cancer Cells. PLoS ONE.

[16] Novak M., Krpan T., Panevska A., Shewell L.K., Day C.J., Jennings M.P., Guella G., Sepčić K. (2020). Binding specificity of ostreolysin A6 towards Sf9 insect cell lipids. Biochim. Biophys. Acta Biomembr..

[17] Panevska A., Hodnik V., Skočaj M., Novak M., Modic Š., Pavlic I., Podržaj S., Zarić M., Resnik N., Maček P. (2019). Pore-forming protein complexes from *Pleurotus* mushrooms kill western corn rootworm and Colorado potato beetle through targeting membrane ceramide phosphoethanolamine. Sci. Rep..

[18] Panevska A., Skočaj M., Križaj I., Maček P., Sepčić K. (2019). Ceramide phosphoethanolamine, an enigmatic cellular membrane sphingolipid. Biochim. Biophys. Acta Biomembr..

[19] Meyer V., Andersen M.R., Brakhage A.A., Braus G.H., Caddick M.X., Cairns T.C., de Vries R.P., Haarmann T., Hansen K., Hertz-Fowler C. (2016). Current challenges of research on filamentous fungi in relation to human welfare and a sustainable bio-economy: A white paper. Fungal Biol. Biotechnol..

[20] Meyer V., Basenko E.Y., Benz J.P., Braus G.H., Caddick M.X., Csukai M., de Vries R.P., Endy D., Frisvad J.C., Gunde-Cimerman N. (2020). Growing a circular economy with fungal biotechnology: A white paper. Fungal Biol. Biotechnol..

[21] de Vries R.P., Riley R., Wiebenga A., Aguilar-Osorio G., Amillis S., Uchima C.A., Anderluh G., Asadollahi M., Askin M., Barry K. (2017). Comparative genomics reveals high biological diversity and specific adaptations in the industrially and medically important fungal genus *Aspergillus*. Genome Biol..

[22] Houbraken J., Kocsubé S., Visagie C.M., Yilmaz N., Wang X.-C., Meijer M., Kraak B., Hubka V., Bensch K., Samson R.A. (2020). Classification of *Aspergillus*, *Penicillium*, *Talaromyces* and related genera (Eurotiales): An overview of families, genera, subgenera, sections, series and species. Stud. Mycol..

[23] Grigoriev I.V., Nikitin R., Haridas S., Kuo A., Ohm R., Otillar R., Riley R., Salamov A., Zhao X., Korzeniewski F. (2014). MycoCosm portal: Gearing up for 1000 fungal genomes. Nucleic Acids Res..

[24] Kjærbølling I., Vesth T.C., Frisvad J.C., Nybo J.L., Theobald S., Kuo A., Bowyer P., Matsuda Y., Mondo S., Lyhne E.K. (2018). Linking secondary metabolites to gene clusters through genome sequencing of six diverse *Aspergillus* species. Proc. Natl. Acad. Sci. USA.

[25] Vesth T.C., Nybo J.L., Theobald S., Frisvad J.C., Larsen T.O., Nielsen K.F., Hoof J.B., Brandl J., Salamov A., Riley R. (2018). Investigation of inter- and intraspecies variation through genome sequencing of *Aspergillus* section Nigri. Nat. Genet..

[26] Kjærbølling I., Vesth T., Frisvad J.C., Nybo J.L., Theobald S., Kildgaard S., Petersen T.I., Kuo A., Sato A., Lyhne E.K. (2020). A comparative genomics study of 23 *Aspergillus* species from section Flavi. Nat. Commun..

[27] Sakaguchi O., Shimada H., Yokota K. (1975). Proceedings: Purification and characteristics of hemolytic toxin from *Aspergillus fumigatus*. Jpn. J. Med. Sci. Biol..

[28] Ebina K., Yokota K., Sakaguchi O. (1982). Studies on toxin of *Aspergillus fumigatus*. XIV. Relationship between Asp-hemolysin and experimental infection for mice. Jpn. J. Med. Mycol..

[29] Wartenberg D., Lapp K., Jacobsen I.D., Dahse H.-M., Kniemeyer O., Heinekamp T., Brakhage A.A. (2011). Secretome analysis of *Aspergillus fumigatus* reveals Asp-hemolysin as a major secreted protein. Int. J. Med. Microbiol..

[30] Rementeria A., López-Molina N., Ludwig A., Vivanco A.B., Bikandi J., Pontón J., Garaizar J. (2005). Genes and molecules involved in *Aspergillus fumigatus* virulence. Rev. Iberoam. Micol..

[31] Nayak A.P., Blachere F.M., Hettick J.M., Lukomski S., Schmechel D., Beezhold D.H. (2011). Characterization of recombinant terrelysin, a hemolysin of *Aspergillus terreus*. Mycopathologia.

[32] Braaksma M., Martens-Uzunova E.S., Punt P.J., Schaap P.J. (2010). An inventory of the *Aspergillus niger* secretome by combining in silico predictions with shotgun proteomics data. BMC Genom..

[33] Lu X., Sun J., Nimtz M., Wissing J., Zeng A.-P., Rinas U. (2010). The intra- and extracellular proteome of *Aspergillus niger* growing on defined medium with xylose or maltose as carbon substrate. Microb. Cell Fact..

[34] Novak M., Čepin U., Hodnik V., Narat M., Jamnik M., Kraševec N., Sepčić K., Anderluh G. (2019). Functional studies of aegerolysin and MACPF-like proteins in *Aspergillus niger*. Mol. Microbiol..

[35] Levin A.M., de Vries R.P., Conesa A., de Bekker C., Talon M., Menke H.H., van Peij N.N.M.E., Wösten H.A.B. (2007). Spatial Differentiation in the Vegetative Mycelium of *Aspergillus niger*. Eukaryot. Cell.

[36] Wösten H.A.B., Moukha S.M., Sietsma J.H., Wessels J.G. (1991). Localization of growth and secretion of proteins in *Aspergillus niger*. J. Gen. Microbiol..

[37] Nitsche B.M., Jørgensen T.R., Akeroyd M., Meyer V., Ram A.F. (2012). The carbon starvation response of *Aspergillus niger* during submerged cultivation: Insights from the transcriptome and secretome. BMC Genom..

[38] Nayak A.P., Green B.J., Janotka E., Hettick J.M., Friend S., Vesper S.J., Schmechel D., Beezhold D.H. (2011). Monoclonal antibodies to hyphal exoantigens derived from the opportunistic pathogen *Aspergillus terreus*. Clin. Vaccine Immunol..

[39] Punt P.J., van den Hondel C.A.M.J.J. (1992). Transformation of filamentous fungi based on hygromycin b and phleomycin resistance markers. Methods Enzymol..

[40] Bagar T., Altenbach K., Read N.D., Benčina M. (2009). Live-Cell Imaging and Measurement of Intracellular pH in Filamentous Fungi Using a Genetically Encoded Ratiometric Probe. Eukaryot. Cell.

[41] Bos C.J., Debets A.J., Swart K., Huybers A., Kobus G., Slakhorst S.M. (1988). Genetic analysis and the construction of master strains for assignment of genes to six linkage groups in *Aspergillus niger*. Curr. Genet..

[42] Mustalahti E., Saloheimo M., Joensuu J.J. (2013). Intracellular protein production in *Trichoderma reesei* (*Hypocrea jecorina*) with hydrophobin fusion technology. N. Biotechnol..

[43] Podobnik B., Stojan J., Lah L., Krasevec N., Seliskar M., Rizner T.L., Rozman D., Komel R. (2008). CYP53A15 of *Cochliobolus lunatus*, a Target for Natural Antifungal Compounds. J. Med. Chem..

[44] Novak M., Sepčić K., Kraševec N., Križaj I., Maček P., Anderluh G., Guella G., Mancini I. (2014). Targeted Lipid Analysis of Haemolytic Mycelial Extracts of *Aspergillus niger*. Molecules.

[45] Hickey P.C., Read N.D. (2009). Imaging living cells of *Aspergillus* in vitro. Med. Mycol..

[46] Cerqueira G.C., Arnaud M.B., Inglis D.O., Skrzypek M.S., Binkley G., Simison M., Miyasato S.R., Binkley J., Orvis J., Shah P. (2014). The *Aspergillus* Genome Database: Multispecies curation and incorporation of RNA-Seq data to improve structural gene annotations. Nucleic Acids Res..

[47] Kersey P.J., Allen J.E., Allot A., Barba M., Boddu S., Bolt B.J., Carvalho-Silva D., Christensen M., Davis P., Grabmueller C. (2018). Ensembl Genomes 2018: An integrated omics infrastructure for non-vertebrate species. Nucleic Acids Res..

[48] Mabey Gilsenan J., Cooley J., Bowyer P. (2012). CADRE: The Central *Aspergillus* Data REpository 2012. Nucleic Acids Res..

[49] Basenko E., Pulman J., Shanmugasundram A., Harb O., Crouch K., Starns D., Warrenfeltz S., Aurrecoechea C., Stoeckert C., Kissinger J. (2018). FungiDB: An Integrated Bioinformatic Resource for Fungi and Oomycetes. J. Fungi.

[50] FungiDB: An Integrated Bioinformatic Resource for Fungi and Oomycetes. https://fungidb.org/fungidb/app.

[51] Jones D.T., Taylor W.R., Thornton J.M. (1992). The rapid generation of mutation data matrices from protein sequences. Bioinformatics.

[52] Kumar S., Stecher G., Li M., Knyaz C., Tamura K. (2018). MEGA X: Molecular Evolutionary Genetics Analysis across Computing Platforms. Mol. Biol. Evol..

[53] FungiDB—Fungal & Oomycete Informatics Resouces. https://beta.orthomcl.org/orthomcl.beta/.

[54] Li L. (2003). OrthoMCL: Identification of Ortholog Groups for Eukaryotic Genomes. Genome Res..

[55] Crabtree J., Angiuoli S.V., Wortman J.R., White O.R. (2007). Sybil: Methods and Software for Multiple Genome Comparison and Visualization. Methods Mol. Biol..

[56] Arnaud M.B., Cerqueira G.C., Inglis D.O., Skrzypek M.S., Binkley J., Chibucos M.C., Crabtree J., Howarth C., Orvis J., Shah P. (2012). The *Aspergillus* Genome Database (AspGD): Recent developments in comprehensive multispecies curation, comparative genomics and community resources. Nucleic Acids Res..

[57] Nierman W.C., Pain A., Anderson M.J., Wortman J.R., Kim H.S., Arroyo J., Berriman M., Abe K., Archer D.B., Bermejo C. (2005). Genomic sequence of the pathogenic and allergenic filamentous fungus *Aspergillus fumigatus*. Nature.

[58] Futagami T., Mori K., Yamashita A., Wada S., Kajiwara Y., Takashita H., Omori T., Takegawa K., Tashiro K., Kuhara S. (2011). Genome announcement: Genome sequence of the white Koji mold *Aspergillus kawachii* IFO 4308, used for brewing the Japanese distilled spirit shochu. Eukaryot. Cell.

[59] Andersen M.R., Salazar M.P., Schaap P.J., Van De Vondervoort P.J.I., Culley D., Thykaer J., Frisvad J.C., Nielsen K.F., Albang R., Albermann K. (2011). Comparative genomics of citric-acid-producing *Aspergillus niger* ATCC 1015 versus enzyme-producing CBS 513.88. Genome Res..

[60] Pel H.J., de Winde J.H., Archer D.B., Dyer P.S., Hofmann G., Schaap P.J., Turner G., de Vries R.P., Albang R., Albermann K. (2007). Genome sequencing and analysis of the versatile cell factory *Aspergillus niger* CBS 513.88. Nat. Biotechnol..

[61] EffectorP: Predicting Fungal Effector Proteins from Secretomes Using Machine Learning. http://effectorp.csiro.au.

[62] Sperschneider J., Dodds P.N., Gardiner D.M., Singh K.B., Taylor J.M. (2018). Improved prediction of fungal effector proteins from secretomes with EffectorP 2.0. Mol. Plant. Pathol..

[63] OCTOPUS: Prediction of Membrane Protein Topology. http://octopus.cbr.su.se/.

[64] Viklund H., Elofsson A. (2008). OCTOPUS: Improving topology prediction by two-track ANN-based preference scores and an extended topological grammar. Bioinformatics.

[65] Phobius a Combined Transmembrane Topology and Signal Peptide Predictor. http://phobius.binf.ku.dk/index.html.

[66] Kall L., Krogh A., Sonnhammer E.L.L. (2007). Advantages of combined transmembrane topology and signal peptide prediction--the Phobius web server. Nucleic Acids Res..

[67] PredGPI GPI-Anchor Predictor. http://gpcr2.biocomp.unibo.it/gpipe/pred.htm.

[68] Pierleoni A., Martelli P., Casadio R. (2008). PredGPI: A GPI-anchor predictor. BMC Bioinform..

[69] PrediSi PREDIction of SIgnal Peptides. http://www.predisi.de/.

[70] Hiller K., Grote A., Scheer M., Munch R., Jahn D. (2004). PrediSi: Prediction of signal peptides and their cleavage positions. Nucleic Acids Res..

[71] PredαTM and PredβTM: Transmembrane Region Predictors. https://www.ki.si/odseki/d01-teoreticni-odsek/laboratorij-za-kemijsko-informatiko/transmembrane-region-predictors/.

[72] Perdih A., Roy Choudhury A., Župerl Š., Sikorska E., Zhukov I., Solmajer T., Novič M. (2012). Structural Analysis of a Peptide Fragment of Transmembrane Transporter Protein Bilitranslocase. PLoS ONE.

[73] Roy Choudhury A., Novič M. (2015). PredβTM: A Novel β-Transmembrane Region Prediction Algorithm. PLoS ONE.

[74] SecretomeP 2.0 Server Prediction of Non-Classical Protein Secretion. http://www.cbs.dtu.dk/services/SecretomeP.

[75] Bendtsen J.D., Jensen L.J., Blom N., von Heijne G., Brunak S. (2004). Feature-based prediction of non-classical and leaderless protein secretion. Protein Eng. Des. Sel..

[76] SignalP-5.0 Server. http://www.cbs.dtu.dk/services/SignalP/.

[77] Almagro Armenteros J.J., Tsirigos K.D., Sønderby C.K., Petersen T.N., Winther O., Brunak S., von Heijne G., Nielsen H. (2019). SignalP 5.0 improves signal peptide predictions using deep neural networks. Nat. Biotechnol..

[78] TargetP-2.0 Server. http://www.cbs.dtu.dk/services/TargetP/.

[79] Almagro Armenteros J.J., Salvatore M., Emanuelsson O., Winther O., von Heijne G., Elofsson A., Nielsen H. (2019). Detecting sequence signals in targeting peptides using deep learning. Life Sci. Alliance.

[80] TMHMM Server v. 2.0. Prediction of Transmembrane Helices in Proteins. http://www.cbs.dtu.dk/services/TMHMM-2.0/.

[81] WOLF PSORT Advanced Computational Tool for Protein Subcellular Localization Prediction. http://www.genscript.com/wolf-psort.html.

[82] Horton P., Park K.-J., Obayashi T., Fujita N., Harada H., Adams-Collier C.J., Nakai K. (2007). WoLF PSORT: Protein localization predictor. Nucleic Acids Res..

[83] Endapally S., Frias D., Grzemska M., Gay A., Tomchick D.R., Radhakrishnan A. (2019). Molecular Discrimination between Two Conformations of Sphingomyelin in Plasma Membranes. Cell.

[84] Anderluh G., Kisovec M., Kraševec N., Gilbert R.J.C. (2014). Distribution of MACPF/CDC Proteins. MACPF/CDC Proteins—Agents of Defence, Attack and Invasion.

[85] McDonagh A., Fedorova N.D., Crabtree J., Yu Y., Kim S., Chen D., Loss O., Cairns T., Goldman G., Armstrong-James D. (2008). Sub-Telomere Directed Gene Expression during Initiation of Invasive Aspergillosis. PLoS Pathog..

[86] Shoji J., Kikuma T., Kitamoto K. (2014). Vesicle trafficking, organelle functions, and unconventional secretion in fungal physiology and pathogenicity. Curr. Opin. Microbiol..

[87] Miura N., Ueda M. (2018). Evaluation of Unconventional Protein Secretion by Saccharomyces cerevisiae and other Fungi. Cells.

[88] Reindl M., Hänsch S., Weidtkamp-Peters S., Schipper K. (2019). A Potential Lock-Type Mechanism for Unconventional Secretion in Fungi. Int. J. Mol. Sci..

[89] Kraševec N., Benčina M., Schmoll M., Dattenböck C. (2016). Gene Expression in Filamentous Fungi: Advantages and Disadvantages Compared to Other Systems. Gene Expression Systems in Fungi: Advancements and Applications, Fungal Biology.

[90] Maraun M., Martens H., Migge S., Theenhaus A., Scheu S. (2003). Adding to ‘the enigma of soil animal diversity’: Fungal feeders and saprophagous soil invertebrates prefer similar food substrates. Eur. J. Soil Biol..

[91] Dubey M., Jensen D.F., Karlsson M. (2020). Functional characterization of the AGL1 aegerolysin in the mycoparasitic fungus *Trichoderma atroviride* reveals a role in conidiation and antagonism. Mol. Genet. Genom..

